# Cadmium uptake kinetics in parts of the seagrass *Cymodocea nodosa* at high exposure concentrations

**DOI:** 10.1186/s40709-018-0076-4

**Published:** 2018-03-06

**Authors:** Paraskevi Malea, Theodoros Kevrekidis, Konstantina-Roxani Chatzipanagiotou, Athanasios Mogias

**Affiliations:** 1 Department of Botany, School of Biology, Aristotle University of Thessaloniki, 54124 Thessaloniki, Greece; 2 Laboratory of Environmental Research and Education, Democritus University of Thrace, Nea Hili, 68100 Alexandroupolis, Greece

**Keywords:** Metal, Accumulation kinetics, Seagrass parts, *Cymodocea nodosa*, Biomonitor, Phytoremediation

## Abstract

**Background:**

Seagrass species have been recommended as biomonitors of environmental condition and as tools for phytoremediation, due to their ability to concentrate anthropogenic chemicals. This study aims to provide novel information on metal accumulation in seagrasses under laboratory conditions to support their use as a tool in the evaluation and abatement of contamination in the field. We investigated the kinetics of cadmium uptake into adult leaf blades, leaf sheaths, rhizomes and roots of *Cymodocea nodosa* in exposure concentrations within the range of cadmium levels in industrial wastewater (0.5–40 mg L^−1^).

**Results:**

A Michaelis–Menten-type equation satisfactorily described cadmium accumulation kinetics in seagrass parts, particularly at 0.5–5 or 10 mg L^−1^. However, an S equation best described the uptake kinetics in rhizomes at 5 mg L^−1^ and roots at 10 and 20 mg L^−1^. Equilibrium concentration and uptake rate tended to increase with the exposure concentration, indicating that seagrass displays a remarkable accumulation capacity of cadmium and reflect high cadmium levels in the surrounding medium. Concerning leaf blades and rhizomes, the bioconcentration factor at equilibrium (range 73.3–404.3 and 14.3–86.3, respectively) was generally lower at higher exposure concentrations, indicating a gradual reduction of available binding sites. Leaf blades and roots accumulated more cadmium with higher rate than sheaths and rhizomes. Uptake kinetics in leaf blades displayed a better fit to the Michaelis–Menten-type equation than those in the remaining plant parts, particularly at 0.5–10 mg L^−1^. A marked variation in tissue concentrations mainly after the steady state was observed at 20 and 40 mg L^−1^, indicative of the stress induced on seagrass cells. The maximum concentrations observed in seagrass parts at 5 and 10 mg L^−1^ were comparatively higher than those previously reported for other seagrasses incubated to similar exposure concentrations.

**Conclusions:**

*Cymodocea nodosa* displays a remarkable cadmium accumulation capacity and reflects high cadmium levels in the surrounding medium. Kinetic models satisfactorily describe cadmium uptake in seagrass parts, primarily in adult leaf blades, at high exposure concentrations, permitting to predict cadmium accumulation in field situations. *Cymodocea nodosa* appeared to be a valuable tool in the evaluation and abatement of cadmium contamination in coastal areas.

## Background

Seagrasses are present in most shallow coastal waters throughout the world, providing a multitude of ecological services and functions. Seagrass beds are highly productive ecosystems, provide habitat and nursery areas for a variety of invertebrates, fish and mammals, and enhance water quality by stabilizing sediments and removing nutrients [1]. Due to their ability to concentrate and retain non-nutrient anthropogenic chemicals in their tissues, seagrass species have been also recommended as biomonitors of environmental condition and as potent tools for phytoremediation [2–4]. The usefulness of seagrasses in the abatement of contamination in coastal areas is corroborated by available data suggesting that these plants appear to be relatively tolerant to many anthropogenic chemicals, particularly more tolerant than other marine flora (macroalgae, kelp, saltmarsh plants, mangroves) and macroinvertebrates [3].

With regard to trace metals, which are of greatest environmental concern [5], several studies, mainly in situ, have examined the accumulation of these contaminants in seagrasses [3, 6]. Laboratory-derived results for metal uptake kinetics are available for about ten seagrass species, for example *Heterozostera tasmanica* [7], *Posidonia oceanica* [8–10] and, most commonly, *Zostera* species [11–13] and *Halophila stipulacea* [14–17]. Metal uptake kinetics only in one or two plant parts, namely leaves or leaves and roots-rhizomes, were investigated in most of these studies [7, 12–17]. Of the trace metals, cadmium, lead and zinc were more commonly used in uptake experiments [7, 9, 11–14, 16]. Recently, the linkage between metal uptake into intermediate-juvenile leaf blades and toxic effects was also examined in a seagrass species (*Cymodocea nodosa*) [18, 19]. However, only in a few of these studies, an attempt was made to describe the uptake patterns by kinetic models [18, 19]. The fit of kinetics data to appropriate models would permit the calculation of uptake kinetic parameters and the prediction of metal accumulation in field situations. Such information could support the use of seagrasses as a tool in the evaluation of contamination in coastal areas or the clean-up of contaminated sites.

The main goal of the present study is to contribute to a better understanding of metal accumulation in seagrasses and to increase the utility of these marine plants as a potent tool in the evaluation and abatement of metal contamination in coastal areas. We assessed the capacity of various parts of *Cymodocea nodosa* to accumulate cadmium from the surrounding medium, as well as the speed of this process. Cadmium was chosen as a contaminant because it is considered highly toxic; it may enter the aquatic environment from various anthropogenic sources, such as zinc, copper and lead mining, various industries, nickel–cadmium batteries and phosphate fertilizers [20]. Cadmium concentrations in the range of 0.1–100 mg L^−1^ are typical in wastewater from several industries (chemical and metal product facilities, leather and tanning processes, electricity and gas production and sanitary industries) [21]. *Cymodocea nodosa* (Ucria) Ascherson along with *Posidonia oceanica* (L.) Delile are the most important and widespread seagrass species in the Mediterranean Sea. *Cymodocea nodosa* is distributed from the intertidal zone to depths of 33–35 m, and can colonize different environmental types, such as open coastal waters, estuaries and coastal lagoons; it is considered as a species with great phenotypic plasticity and a high capacity to adapt to environmental variability and thereby to colonize new substrates [22–24]. *Cymodocea nodosa* is also considered as a good biomonitor for trace elements, with leaves being the best part for the determination of element loads [25]. In addition, microtubule integrity in leaf cells of *C. nodosa* is regarded as an early marker of metal-induced stress [18, 19, 26]. Under laboratory conditions, we investigated the kinetics of cadmium uptake into adult leaf blades, leaf sheaths, rhizomes and roots of *Cymodocea nodosa* exposed to concentrations of cadmium ranging from 0.5 to 40 mg L^−1^; the uptake kinetics data were fitted to different models.

## Methods

### Plant collection

*Cymodocea nodosa* was collected from the eastern coast of the Gulf of Thessaloniki, Northern Aegean Sea at the Viamyl site (site V, 40°33′N, 22°58′E). At this site, *C. nodosa* grows from 0.4 m to around 2 m depth, forming a continuous monospecific meadow. Leaf, rhizome and root biomass displays an annual mean value of approx. 60, 122 and 65 g dry wt m^−2^, respectively [27]. Leaf biomass and leaf blade length display an almost unimodal annual pattern; both markedly increase from March to July–August attaining a maximum value of approx. 150 g dry wt m^−2^ and 542.2 mm, respectively, while rhizome biomass and root biomass peak in mid or late summer and in mid autumn to early winter attaining a maximum value of approx. 225 and 125 g dry wt m^−2^, respectively ([27], unpublished data).

Plants were collected at the site V at 0.7–1.0 m depth in July 2011 with a 20 cm diameter acrylic corer, which penetrated to a depth of 30 cm; all the above- and below-substrate plant material rooted within this area was collected. All plants were rinsed in seawater at the collection site and transported to the laboratory in plastic containers containing seawater.

### Treatments

Fresh green plants without epiphytes were kept for 24 h in seawater under laboratory conditions in order to equilibrate. Plants were incubated in plastic aquaria containing 10 L of cadmium sulphate hydrate (3CdSO_4_·8H_2_O 98.7%, insoluble matter ≤ 0.005%, chloride ≤ 0.001%, total nitrogen ≤ 0.0005%, Ca ≤ 0.005%, Cu ≤ 0.0005%, Fe ≤ 0.0005%, K ≤ 0.01%, Na ≤ 0.005%, Pb ≤ 0.002%, Zn ≤ 0.002%; lot number: 1.02027.0100; Merck, Darmstadt, Germany) dissolved in filtered (Whatman GF/C) seawater at one of the following cadmium concentrations: 0.5, 5, 10, 20 and 40 mg L^−1^, corresponding to 4.44, 44.48, 88.95, 177.94 and 355.88 μΜ, respectively. Plant material of about 12.5 g dry wt, including about 70 leaf shoots and the corresponding rhizomes and roots, was incubated in each aquarium; no sediment and no complexing agents were added. The seawater used for the experiments was also collected from the sampling site. The seawater used in the experiments had salinity 36.7 psu, pH 7.9, dissolved O_2_ 5.88 mg L^−1^, N-NO_2_^−^ 0.02 μΜ, N-NO_3_^−^ 0.37 μΜ, Ν-NH_4_^+^ 0.43 μΜ and cadmium 0.267 μg L^−1^. The media in the aquaria were changed every 2 days in order to maintain the original levels. The aquaria were aerated constantly using aquarium pumps and covered with plastic film (Sanitas, Sarantis S.A., Athens, Greece) in order to prevent evaporation. The experiments were conducted under a constant 16:8 h day:night regime at ambient temperature of 21 ± 1 °C and irradiance of 120 μmοl m^−2^ s^−1^. After 0, 3, 5, 7 and 9 days, at least three samples, each one including 3 or 4 leaf shoots (about 10 leaves, approx. 270 mg dry wt) and the corresponding rhizomes (approx. 210 mg dry wt) and roots (approx. 140 mg dry wt), were removed at random from each aquarium. Plant material was separated into leaves, rhizomes and roots. Seagrass leaves were characterized as adult or intermediate-juvenile; adult leaves, which accounted for about 85% of the total leaf material, were used for cadmium analysis. Adult leaf material was further partitioned into leaf blades and leaf sheaths. Leaf age estimation was based on the morphological features of the sheath; leaves with a well-developed sheath that encompassed other leaves in their interior were classed as adults [28]. Similar procedures have been used in previous studies [14–16].

### Cadmium determination

The material from each plant compartment (adult leaf blades, leaf sheaths, rhizomes and roots) of the samples from a single aquarium collected on the same day was pooled. The pooled samples were washed in double-distilled water, dried to a constant weight (60 °C) and ground in an agate mill. Three subsamples of each powdered sample were wet digested in HNO_3_/HClO_4_ (4:1) at 50 °C for 1 h and then at 130 °C for 3 h. Similar methods have been frequently used in previous studies [14–16, 18]. Cadmium concentrations were measured using graphite furnace atomic absorption spectrophotometry (AAS, AANALYST 700 Perkin-Elmer). Pro-analysis grade reagents were used and reagent blanks were run concurrently. Standards were prepared by serial dilution of stock solutions. The accuracy of the technique was checked by analysis of standard sea lettuce reference material (*Ulva lactuca* no 279, Community Bureau of Reference BCR, Brussels, Belgium); one sample of the standard reference material was included in each analytical batch. Results were in agreement with certified values (certified value, mean ± SD: 0.274 ± 0.032 μg g^−1^ dry wt; measured value, mean ± SD: 0.280 ± 0.020 μg g^−1^ dry wt; recovery: 102%).

### Data analysis

Cadmium accumulation kinetics were fitted to a version of the Michaelis–Menten equation; this Michaelis–Menten-type equation (Eq. 1) is frequently used in metal kinetic studies, where C represents the tissue metal concentration reached in time *t*, C_max_ the maximum or saturation concentration, and *K*_*m*_ the time taken to reach half of the value of C_max_ [29–31].

1 $${\text{C}} = {{\left( {{\text{C}}_{\text{max} } \times t} \right)} \mathord{\left/ {\vphantom {{\left( {{\text{C}}_{\hbox{max} } \times t} \right)} {\left( {K_{m} + t} \right)}}} \right. \kern-0pt} {\left( {K_{m} + t} \right)}}$$

The data were analyzed using IBM Statistics SPSS^®^ 19 (New York, NY, USA), by means of nonlinear regression, and with sequential quadratic programming as the estimation method [31]. The rate of the initial uptake (found by dividing half of the value of C_max_ by *K*_*m*_) was also estimated.

When the fit to Eq. 1 was not significant at the 0.01 level, the data were fitted to different regression models (linear, Eq. 2; logarithmic, Eq. 3; inverse, Eq. 4; exponential, Eq. 5; power, Eq. 6; S, Eq. 7), also using IBM Statistics SPSS^®^ 19; in each case, the model that provided the best fit (i.e., at the highest significance level) was chosen [31].

2 $${\text{C}} = \alpha + b \times t$$

3 $${\text{C}} = \alpha + b \times \ln \left( {t + 1} \right)$$

4 $${\text{C}} = \alpha + \, \left[ {b{{} \mathord{\left/ {\vphantom {{} {\left( {t + 1} \right)}}} \right. \kern-0pt} {\left( {t + 1} \right)}}} \right]$$

5 $${\text{C}} = \alpha \times e^{b\; \times \;t}$$

6 $$\text{lnC} = \ln \alpha + b \times \ln \left( {t + 1} \right)$$

7 $$\text{lnC} = \alpha + \left[ {{b \mathord{\left/ {\vphantom {b {\left( {t + 1} \right)}}} \right. \kern-0pt} {\left( {t + 1} \right)}}} \right]$$

In order for the information on accumulation kinetics to be completed, the time required to reach equilibrium (T_eq_), the equilibrium concentration (C_eq_) and the mean rate of uptake (V_c_) were calculated. C_eq_ was estimated from the equation used in each case as the tissue concentration at which the daily increase in concentration was less than 1% of that of the previous day, T_eq_ was estimated as the time required to reach C_eq_, and V_c_ by dividing C_eq_ by T_eq_ [29, 31]. Bioconcentration factor (BCF) at equilibrium was also calculated (Eq. 8), where C_eq_ is the equilibrium concentration, C_i_ the initial tissue metal concentration (at day 0) and C_w_ the metal concentration in the water [31, 32].

8 $${\text{BCF}}\, = {{\left( {{\text{C}}_{\text{eq}} \;{-}\;{\text{C}}_{\text{i}} } \right)} \mathord{\left/ {\vphantom {{\left( {{\text{C}}_{\text{eq}} \;{-}\;{\text{C}}_{\text{i}} } \right)} {{\text{C}}_{\text{w}} }}} \right. \kern-0pt} {{\text{C}}_{\text{w}} }}\,$$

The Wilcoxon matched-pairs signed-rank test was used to compare experimental metal concentrations and uptake parameters in different plant parts. Spearman’s rank correlation coefficient (ρ) was calculated to identify correlations.

## Results

The initial concentrations of cadmium (0 day, mean ± standard error) in adult leaf blades, leaf sheaths, rhizomes and roots of *C. nodosa* were 1.773 ± 0.188, 1.066 ± 0.258, 0.655 ± 0.036 and 0.258 ± 0.008 μg g^−1^ dry wt, respectively.

**Fig. 1:**
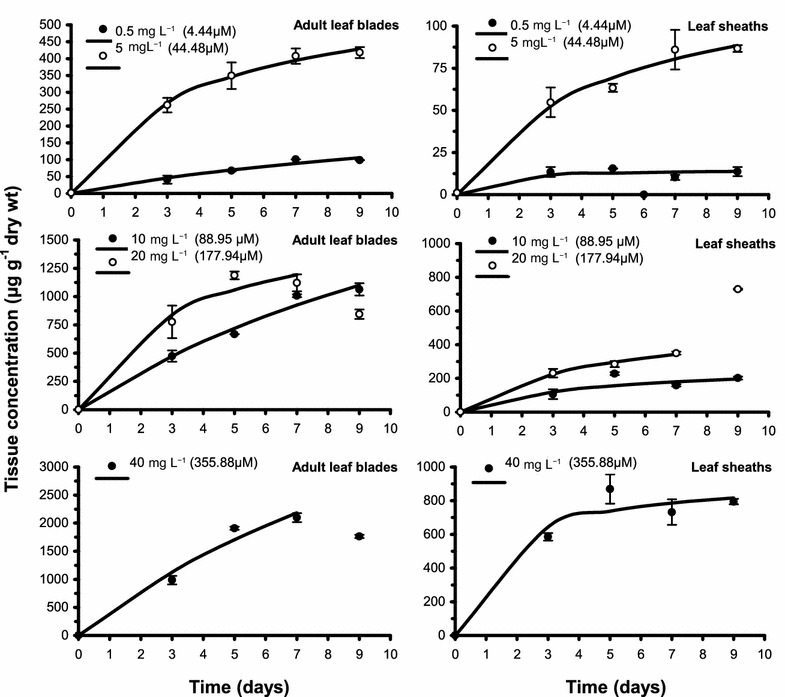
Kinetics of cadmium accumulation in adult leaf blades and leaf sheaths of *Cymodocea nodosa* at different concentrations of cadmium in water. Values plotted are mean tissue concentration ± standard error (n = 3); bold lines are the accumulation kinetics calculated using a Michaelis–Menten-type equation

**Fig. 2:**
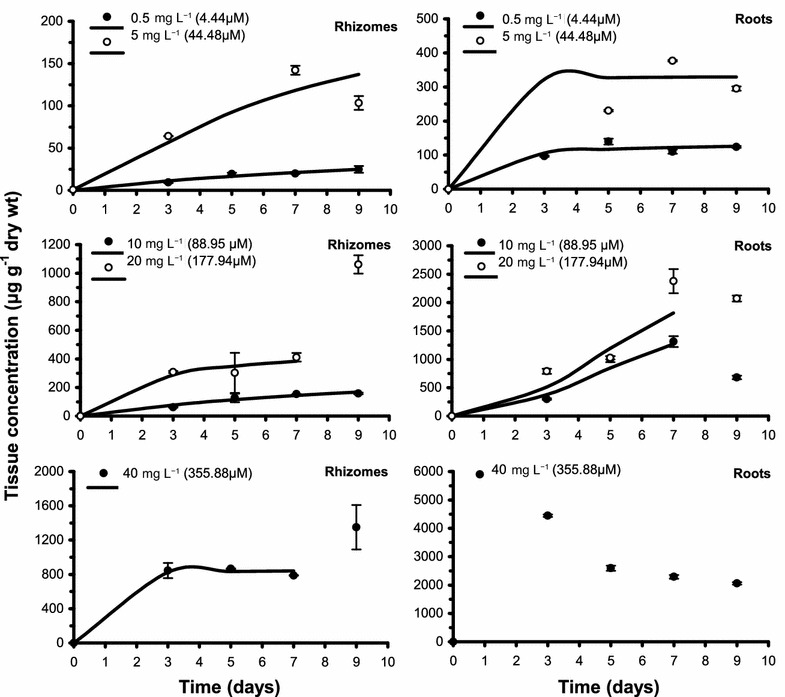
Kinetics of cadmium accumulation in rhizomes and roots of *Cymodocea nodosa* at different concentrations of cadmium in water. Values plotted are mean tissue concentration ± standard error (n = 3); bold lines are the accumulation kinetics calculated using a Michaelis–Menten-type equation (rhizomes at 0.5, 10, 20 and 40 mg L^−1^ and roots at 0.5 and 5 mg L^−1^) or an S equation (rhizomes at 5 mg L^−1^ and roots at 10 and 20 mg L^−1^)

The accumulation kinetics of cadmium in *C. nodosa* parts were rapid in the first 3–5 days of exposure; this initial rapid accumulation was generally followed by a slower accumulation phase and/or a steady state (Figs. 1 and 2). A variation on the last incubation day was observed at the higher exposure concentrations, particularly a marked increase in leaf sheaths at 20 mg L^−1^ and rhizomes at 20 and 40 mg L^−1^, and, on the contrary, a marked decrease in adult leaf blades at 20 and 40 mg L^−1^ and roots at 10 and 20 mg L^−1^. In addition, the initial rapid accumulation in roots at 40 mg L^−1^ was followed by a marked decrease after the third day of exposure.

For each incubation day, tissue cadmium concentrations generally tended to increase with the exposure concentration (C_w_; Figs. 1 and 2); in particular, a significant and positive correlation was found between tissue metal content and C_w_ for all plant parts and all incubation times (ρ = 0.9, *p* < 0.05 or ρ = 1.0, *p* < 0.001; Table 1). Cadmium concentrations reached during the incubation period generally differed among plant parts (Figs. 1 and 2); in particular, adult leaf blades and roots displayed significantly higher cadmium concentrations than either leaf sheaths or rhizomes, when both the lower exposure concentrations (0.5–10 mg L^−1^) and the full set of exposure concentrations (0.5–40 mg L^−1^) were examined (Wilcoxon test, *p* < 0.01 and 0.001, respectively).

**Table 1:** Spearman’s rank correlation coefficient values between exposure concentration and experimental tissue cadmium concentration (C) at each incubation day

Variables	Blades	Sheaths	Rhizomes	Roots
C at day 3	1.0***	1.0***	0.9*	0.9*
C at day 5	1.0***	1.0***	1.0***	1.0***
C at day 7	1.0***	1.0***	1.0***	0.9*
C at day 9	0.9*	1.0***	1.0***	0.9*

* *p *< 0.05; *** *p *< 0.001

The fit of the kinetics of cadmium accumulation in adult leaf blades, leaf sheaths and rhizomes at the 0.5–10 mg L^−1^ treatments and in roots of *C. nodosa* at the 0.5 and 5 mg L^−1^ treatments to the Michaelis–Menten-type equation was generally significant (r^2^: 0.812–0.997, *p* < 0.01 or 0.001; Figs. 1 and 2, Table 2). Metal uptake kinetics in adult leaf blades generally displayed a better fit (i.e., at a higher significance level) to the Michaelis–Menten-type equation, compared to those in leaf sheaths and roots. Concerning rhizomes at 5 mg L^−1^, the fit to the Michaelis–Menten-type equation was not significant at the 0.01 level; in this case, an S equation was found to best describe the uptake kinetics (r^2^: 0.993, *p* < 0.01; Fig. 2, Tables 3 and 4).

**Table 2:** Kinetics of cadmium accumulation in parts of *Cymodocea nodosa* exposed to different concentrations of cadmium in water

Exposure concentration (mg L^−1^)					
	0.5	5	10	20	40
Blades					
C_max_	306.5 (± 194.8)	611.2 (± 39.0)	3289.5 (± 1438.7)	1748.5 (± 559.9)^a^	7386.1 (± 6749.5)^a^
*K*_*m*_	17.1 (± 15.3)	3.8 (± 0.6)	17.9 (± 10.9)	3.3 (± 2.6)	16.7 (± 20.4)
C_max_/(2 × *K*_*m*_)	9.0	80.4	91.9	264.9	221.1
r^2^	0.968***	0.997***	0.986***	0.971**	0.975**
Sheaths					
C_max_	13.5 (± 3.0)	134.8 (± 22.8)	272.8 (± 132.6)	568.4 (± 73.3)^a^	942.6 (± 180.6)
*K*_*m*_	0.1 (± 1.1)	4.7 (± 1.8)	3.1 (± 4.3)	4.6 (± 1.3)	1.4 (± 1.3)
C_max_/(2 × *K*_*m*_)	67.5	14.3	44.0	61.8	336.6
r^2^	0.881**	0.985***	0.837**	0.997***	0.950**
Rhizomes					
C_max_	63.7 (± 33.9)		405.2 (± 204.8)	514.2 (± 158.2)^a^	852.0 (± 82.0)^a^
*K*_*m*_	14.1 (± 11.2)		12.7 (± 9.8)	2.4 (± 2.2)	0.1 (± 0.4)
C_max_/(2 × *K*_*m*_)	2.2		16.0	107.1	4260.0
r^2^	0.968***		0.968***	0.965**	0.992**
Roots					
C_max_	138.8 (± 27.8)	333.3 (± 106.0)			
*K*_*m*_	0.9 (± 1.2)	0.1 (± 1.5)			
C_max_/(2 × *K*_*m*_)	77.1	1666.5			
r^2^	0.937**	0.812**			

The exposure concentrations of 0.5, 5, 10, 20 and 40 mg L^−1^ correspond to 4.44, 44.48, 88.95, 177.94 and 355.88 μM, respectively

The fits correspond to a Michaelis–Menten-type equation: C = (C_max_ × *t*)/(*K*_*m* _+ *t*)

C, tissue concentration (μg g^−1^ dry wt) reached in a given time; C_max,_ maximum tissue concentration; *K*_*m*,_ time (in days) to reach half of the value of C_max_; *t*, time (in days); standard errors are given in parentheses

** *p *< 0.01; *** *p *< 0.001

^a^Only the initial four points were fitted

**Table 3:** Regression models tested in those cases in which the Michaelis–Menten-type model did not provide a satisfactory fit to the uptake kinetics data

	Exposure concentration (mg L^−1^)	Model	r^2^	F	df_1_	df_2_	Significance
Rhizomes	5	Linear	0.778	7.003	1	2	0.118
		Logarithmic	0.868	13.130	1	2	0.068
		Inverse	0.815	8.811	1	2	0.097
		Exponential	0.702	4.717	1	2	0.162
		Power	0.917	22.175	1	2	0.042
		S	0.993	292.077	1	2	0.003
Roots	10	Linear	0.945	34.576	1	2	0.028
		Logarithmic	0.802	8.106	1	2	0.104
		Inverse	0.639	3.533	1	2	0.201
		Exponential	0.825	9.429	1	2	0.092
		Power	0.973	73.230	1	2	0.013
		S	0.998	1200.35	1	2	0.001
	20	Linear	0.903	18.599	1	2	0.050
		Logarithmic	0.749	5.972	1	2	0.134
		Inverse	0.602	3.021	1	2	0.224
		Exponential	0.792	7.606	1	2	0.110
		Power	0.954	41.675	1	2	0.023
		S	0.997	581.401	1	2	0.002

The exposure concentrations of 5, 10 and 20 mg L^−1^ correspond to 44.48, 88.95 and 177.94 μM, respectively

**Table 4:** Kinetics of cadmium accumulation in parts of *Cymodocea nodosa* exposed to different concentrations of cadmium in water

	Exposure concentration (mg L^−1^)		
	5	10	20
Rhizomes			
*α*	5.512 (± 0.180)		
*b*	− 5.909 (± 0.346)		
r^2^	0.993**		
Roots			
*α*		8.366 (± 0.148)^a^	8.980 (± 0.225)^a^
*b*		− 9.745 (± 0.281)	− 10.313 (± 0.428)
r^2^		0.998**	0.997**

The exposure concentrations of 5, 10 and 20 mg L^−1^ correspond to 44.48, 88.95 and 177.94 μM, respectively

The fits correspond to an S equation: lnC = *α* + [*b*/(*t *+ 1)]

C, tissue concentration (μg g^−1^ dry wt) reached in a given time; α and b constants; *t*, time (in days); standard errors are given in parentheses

** *p *< 0.01

^a^Only the initial four points were fitted

The fit of cadmium uptake kinetics in adult leaf blades, leaf sheaths and rhizomes at the higher exposure concentrations (20 and 40 mg L^−1^) to the Michaelis–Menten-type equation was also significant (r^2^: 0.950–0.997, *p* < 0.01 or 0.001; Figs. 1 and 2, Table 2); as for roots at the 10 and 20 mg L^−1^ treatments, an S equation best described the uptake kinetics (r^2^: 0.997–0.998, *p* < 0.01; Fig. 2, Tables 3 and 4). However, in most of these cases, the initial four points only were used because of the marked variation in tissue metal content on the last day of incubation. The kinetics of cadmium uptake in roots at 40 mg L^−1^ were not fitted to any model because of the marked decrease in tissue metal content after the third day of incubation (Fig. 2).

The values of the uptake parameters obtained for *C. nodosa* parts are shown in Tables 2 and 5. The values of the maximum concentration (C_max_) and the equilibrium concentration (C_eq_) generally tended to increase with C_w_; in particular, a significant and positive correlation was found between C_max_ and C_w_ for adult leaf blades, leaf sheaths and rhizomes, and between C_eq_ and C_w_ for adult leaf blades, leaf sheaths and roots (ρ = 0.9, *p* < 0.05 or ρ = 1.0, *p* < 0.001; Table 5). The values of the rate of initial uptake (C_max_/(2 × *K*_*m*_)) and the mean rate of the uptake (V_c_) also generally tended to increase with C_w_ (Tables 2 and 5); a significant and positive correlation was particularly found between C_max_/(2 × *K*_*m*_) and C_w_ for adult leaf blades and rhizomes (ρ = 0.9, *p* < 0.05 and ρ = 1.0, *p* < 0.001, respectively), and between V_c_ and C_w_ for all plant parts (ρ = 1.0, *p* < 0.001; Table 6). Thereby, higher C_max_ and C_eq_ values were reached with higher uptake rates.

**Table 5:** Equilibrium concentration, (C_eq_ in μg g^−1^ dry wt), time to reach equilibrium (T_eq_, in days), mean rate of uptake (V_c_, in concentration/days) and bioconcentration factor at equilibrium (BCF)

Exposure concentration (mg L^−1^)					
	0.5	5	10	20	40
Blades					
C_eq_	203.9	504.7	2155.0	1467.3^a^	4908.6^a^
T_eq_	34	18	34	17	33
V_c_	6.0	28.0	63.4	86.3	148.7
BCF	404.3	100.6	215.3	73.3	122.7
Sheaths					
C_eq_	11.7	109.1	228.5	457.6^a^	836.2
T_eq_	14	20	16	19	11
V_c_	0.8	5.5	14.3	24.1	76.0
BCF	21.3	21.6	22.7	22.8	20.9
Rhizomes					
C_eq_	43.8	193.6	144.0	439.0^a^	824.5^a^
T_eq_	31	22	7	14	3
V_c_	1.4	8.8	20.6	31.4	274.8
BCF	86.3	38.6	14.3	21.9	20.6
Roots					
C_eq_	126.2	322.5	3138.9^a^	5694.9^a^	
T_eq_	9	3	29	29	
V_c_	14.0	107.5	108.2	196.4	
BCF	251.9	64.4	313.9	284.7	

The exposure concentrations of 0.5, 5, 10, 20 and 40 mg L^−1^ correspond to 4.44, 44.48, 88.95, 177.94 and 355.88 μM, respectively

^a^Only the initial four points were fitted

**Table 6:** Spearman’s rank correlation coefficient values between exposure concentration uptake parameters; n = 5

Variables	Blades	Sheaths	Rhizomes	Roots
C_max_	0.9*	1.0***	1.0***	
K_m_	− 0.3^ns^	0.1^ns^	− 1.0***	
C_max_/(2 × K_m_)	0.9*	0.4^ns^	1.0***	
C_eq_	0.9*	1.0***	0.8^ns^	1.0***
T_eq_	− 0.41^ns^	− 0.3^ns^	− 0.9*	0.737^ns^
V_c_	1.0***	1.0***	1.0***	1.0***
BCF	− 0.5^ns^	0.0^ns^	− 0.7^ns^	0.6^ns^

For abbreviations, see Tables 2 and 5

^ns^Non significant; * *p *< 0.05; *** *p *< 0.001

The values of the time taken to reach half of the C_max_ (*K*_*m*_) and the time taken to reach equilibrium (T_eq_) obtained for adult leaf blades displayed no clear pattern with increasing C_w_; in leaf sheaths, these parameters displayed their lowest values at the lowest and the highest exposure concentration respectively, while in roots, T_eq_ displayed its highest value at the higher exposure concentrations. As for rhizomes, *K*_*m*_ and T_eq_ generally tended to decrease with increasing C_w_ (ρ = − 1.0, *p* < 0.001 and ρ = − 0.9, *p* < 0.05, respectively; Tables 2, 5 and 6).

Comparable BCF values were obtained for leaf sheaths at all of the treatments (range 20.9–22.8); as for roots, BCF values (range 64.4–313.9) showed no clear trend with increasing C_w_, while as for adult leaf blades and rhizomes, BCF values (range 73.3–404.3 and 14.3–86.3, respectively) were generally lower at higher exposure concentrations (Tables 5 and 6).

C_eq_ and BCF values obtained for adult blades were significantly higher than those obtained for leaf sheaths and rhizomes; V_c_ values estimated for adult leaf blades and rhizomes were also significantly higher than those estimated for leaf sheaths (Wilcoxon test, *p* < 0.05). In addition, at each exposure concentration, C_eq_, V_c_ and BCF generally had the highest values in adult leaf blades and roots, and the lowest values in leaf sheaths and rhizomes (Table 5).

## Discussion

The initial concentrations of cadmium in adult leaf blades, leaf sheaths, rhizomes and roots of *Cymodocea nodosa* collected for the experiments were within the range of cadmium concentrations earlier measured in *C. nodosa* parts from various localities along Mediterranean coasts (see review in [28]). The maximum experimental concentration observed in adult leaf blades of *C. nodosa* (101.3 μg g^−1^ dry wt) at the lowest exposure concentration studied (0.5 ml L^−1^) was within the wide range of reported cadmium concentrations in leaves of seagrass species (0.1–266 μg g^−1^ dry wt) from various geographical locations (see review in [3]).

Leaf blades and roots are the main sites of ionic uptake in seagrasses [33, 34] and metal uptake is considered to follow two pathways-from surrounding water to leaves and then to rhizomes, or from interstitial water into roots to rhizomes and leaves [35]. Translocation of cadmium within a seagrass species (*Zostera marina*) has been previously demonstrated in laboratory experiments; in particular, both a bidirectional translocation and a translocation from leaves to root-rhizomes have been reported [36, 37].

The uptake kinetics of cadmium into parts of *C. nodosa* at the lower exposure concentrations examined (0.5–5 or 10 mg L^−1^), namely at concentrations within the range of cadmium concentrations in industrial wastewater [21], generally displayed a similar pattern: an initial rapid accumulation was followed by a slower accumulation phase and an equilibrium state. This pattern is consistent with previous observations concerning the accumulation of cadmium into parts of marine angiosperms [7, 11, 14, 18, 36]. A Michaelis–Menten-type equation satisfactorily described cadmium accumulation kinetics in most cases, while an S equation best described the uptake kinetics in rhizomes at 5 mg L^−1^ and roots at 10 mg L^−1^, both permitting to calculate uptake parameters.

The observed accumulation kinetics was most probably the net result of several different processes. A potential release by the seagrass into the medium of organic ligands, capable to complex dissolved metals, may have played a role in controlling the cadmium uptake [38–40]. Cadmium accumulation may involve a combination of adsorption onto the outer cell wall and uptake into the cells; the steady state may correspond to an equilibrium attained between the metal in the medium and the metal bound on the cell surface, while intracellular uptake may include both a diffusion across the plasma membrane into the protoplasm and an active accumulation of metal within the plant cells [29, 32, 41, 42]. Hence, cadmium is expected to be accumulated in different cellular compartments. In addition, cadmium accumulation, particularly in leaf sheaths and rhizomes, may have resulted from both a direct uptake from the surrounding medium and some internal transport.

Tissue cadmium concentrations reached at each incubation time at the lower exposure concentrations (0.5–5 or 10 mg L^−1^) and, thereby, uptake parameters too, appeared to be a function of cadmium concentration in water. The maximum concentration and the equilibrium concentration, as well as the rate of initial uptake and the mean rate of uptake respectively, generally tended to increase with the exposure concentration, indicating that *C. nodosa* parts display a remarkable absorption capacity of cadmium and an abundance of cell wall or intracellular binding sites [43]. This finding also indicates that cadmium in *C. nodosa* is correlated with that in the surrounding medium over a wide range of exposure concentrations [30]. However, as for adult leaf blades and rhizomes, the bioconcentration factor at equilibrium generally displayed lower values at higher exposure concentrations, suggesting a reduction of available binding sites [7] and, thereby, a lower sensitivity in detecting high levels of cadmium contamination [31].

Cadmium concentrations reached during the incubation period at the lower exposure concentrations (0.5–5 or 10 mg L^−1^) and, thereby, uptake kinetic parameters too, appeared to differ to some extent among *C. nodosa* parts, indicating within-plant differences in cadmium accumulation capacity. Adult leaf blades and roots, which present the highest surface/volume ratio and are the main sites of ionic uptake [33–35, 43], generally accumulated more cadmium with higher uptake rates than leaf sheaths and rhizomes, in which, as noted earlier, metal accumulation may have resulted from both a direct uptake from the surrounding medium and some internal transport. In addition, cadmium uptake kinetics in adult leaf blades generally displayed a better fit to the Michaelis–Menten-type equation, compared to those in the remaining plant parts. Variability in the distribution pattern of cadmium among *C. nodosa* parts might be expected in field situations due to differences in the bioavailability of this element in the water column and sediment. Nevertheless, our findings are consistent with most of the previous field observations, whereby *C. nodosa* leaves and roots accumulated the highest cadmium loads and rhizomes the lowest ones (see review in [28]); in particular, at the same sampling area (Viamyl site), *C. nodosa* displayed over the year significantly lower concentrations of cadmium in rhizome parts than in blades, sheaths and roots [28]. A comparison of the experimental cadmium concentrations reached in adult leaf blades of *C. nodosa* during the incubation period with the corresponding concentrations in intermediate-juvenile leaf blades of this seagrass species incubated to the same exposure concentrations [18] reveals that adult leaf blades accumulate more cadmium than intermediate and juvenile ones; this finding could be explained by a synthesis of more binding sites over time [44, 45].

The above findings suggest that *C. nodosa* could be considered as a potent tool for the assessment of cadmium levels of 0.5–5 or 10 mg L^−1^ in the surrounding medium in coastal areas after an accidental discharge of untreated metal-bearing industrial effluents or in inshore wastewater treatment areas. Considering both our data and operational criteria (e.g. ease of sampling), adult leaf blades appear to be the most suited plant part for the determination of cadmium levels in the surrounding environment. The above findings also suggest that *C. nodosa* could be considered as a valuable tool for the treatment of cadmium from industrial effluents or in the abatement of cadmium contamination in coastal environments receiving untreated industrial effluents, when exposure concentrations range from 0.5 to 5 or 10 mg L^−1^. For instance, contaminant amounts could be removed by harvesting mainly seagrass adult leaves when the equilibrium state in metal accumulation is achieved [46].

A comparison of the maximum experimental cadmium concentrations observed in parts of *C. nodosa* with those previously reported for the respective parts of other seagrass species incubated to similar concentrations of cadmium in seawater corroborates the finding that *C. nodosa* displays a remarkable accumulation capacity of cadmium. For instance, the maximum experimental cadmium concentrations in rhizomes and roots of *C. nodosa* (142.1 and 405.6 μg g^−1^ dry wt, respectively) at the 5 mg L^−1^ treatment were higher than those in rhizomes and roots of *Zostera marina* (approx. 56.0 and 320.5 μg g^−1^ dry wt, respectively) exposed for 19 days to seawater containing 5.62 mg L^−1^ cadmium [11]; the maximum experimental cadmium concentration in adult leaf blades of *C. nodosa* (1063.6 μg g^−1^ dry wt) at the 10 mg L^−1^ treatment was also higher than that in leaves of *Halophila stipulacea* (721 μg g^−1^ dry wt) incubated for 16 days in seawater containing cadmium in concentration of 11.24 mg L^−1^ [14]. In addition, the maximum experimental cadmium concentration in adult leaf blades of *C. nodosa* (1063.6 μg g^−1^ dry wt) at the 10 mg L^−1^ treatment was higher compared to the respective one in leaves of the submerged brackish-water angiosperm *Ruppia maritima* (772.0 μg g^−1^ dry wt) exposed to lagoon water (8.6 psu salinity) also containing cadmium in concentration of 10 mg L^−1^ [47], despite a decreased cadmium accumulation with increasing salinity is expected due to the decreased availability of cadmium in the medium because of the complexes formed between chloride and metal [48].

The marked variation in tissue cadmium concentrations mainly observed at the last day of incubation at the higher exposure concentrations studied (20–40 mg L^−1^) could be related to the stress induced on seagrass cells. A potential explanation for the marked decrease in adult leaf blade and root cadmium concentrations is a release of accumulated metal quantities to the exterior [29]. Seagrass cells may possess active efflux systems for non-essential elements, as observed for cadmium in marine diatoms [42], to minimize the toxic impact of metal exposure [49]. A potential release (leakage) of cadmium by tissues of a seagrass species (*Zostera marina*) has been previously reported by Brinkhuis et al. [36]. This marked decrease could be also ascribed to an extended internal transport of accumulated metal quantities from roots and leaf blades towards to rhizomes and leaf sheaths; this interpretation is corroborated by the observation that leaf sheath and rhizome cadmium concentrations markedly increased at the last day of exposure at the 20 mg L^−1^ treatment (as for sheaths) or at both the 20 and 40 mg L^−1^ treatments (as for rhizomes). This marked increase in leaf sheath and rhizome cadmium concentrations could also be ascribed to an increase in available binding sites due to potential cell deterioration [11, 12, 14].

## Conclusions

*Cymodocea nodosa* displays a remarkable accumulation capacity of cadmium, varying among plant parts and reflects high cadmium levels in the surrounding medium. Kinetic models, usually a Michaelis–Menten-type equation, satisfactorily describe cadmium uptake patterns in plant parts, primarily in adult leaf blades, at exposure concentrations within the range of cadmium levels in industrial wastewater, permitting to calculate uptake parameters and to predict cadmium accumulation in field situations. The data presented contribute to a better understanding of metal accumulation in seagrasses and highlight the usefulness of *C. nodosa* as a biomonitor of cadmium contamination and as a valuable tool for phytoremediation, when exposure concentrations range from 0.5 to 5 or 10 mg L^−1^.
